# The Waters of New York Harbor

**Published:** 1888-03

**Authors:** 


### *THE WATERS OF NEW YORK HARBOR.*

Dr. J. J. Kinyoun, Assistant Surgeon United States Marine Hospital Service, has recently made a careful examination of the waters of New York harbor, showing conclusively that the germs of cholera and many other infectious diseases are capable of living and multiplying indefinitely therein.

The cities discharging sewage into New York bay have a population of 3,000,000 people. Specimens were obtained at the incoming tide from the Narrows, from the side of the steamship *Britannia* lying in quarantine, from near Hoffman's island and Swinburne island. A chemical examination demonstrated that the amount of organic impurity was very great, and about the same in each of these samples. The samples were collected in sterilized flasks; and in order to ascertain the nature of the bacteria contained therein, and determine how long they would support life of the different micro-organisms, the following experiments were made:

"Plate cultivations were made from each of the different specimens, and, at the end of five days, had developed colonies of bacteria. Examination showing the number of micro-organisms:

"Narrows, 4,500 to cubic centimeter; *Britannia* anchorage, 10,200 to cubic centimeter; Hoffman island, 9,600 to cubic centimeter; Swinburne island, 11,700 to cubic centimeter.

"The micro-organisms found in each were several varieties of micrococci and one of a large bacillus. These were transferred to cultivation-tubes for further observation. On November 12th, test-tubes, partly filled with sea-water, were thoroughly sterilized and inoculated in the usual manner, with pure cultivations of the spirilla of Asiatic cholera, and also of Finkler and Prior. Cultivation-tubes were inoculated from the water from day to day, for the purpose of determining the longevity of the growths. During the first five days, the water seemed to exert a slight inhibitory influence over their development. It was further observed that until January 20th, a period of sixty-nine days, the characteristic growth of the spirillum of cholera Asiatica could be produced in peptone gelatine. That of Finkler and Prior has a yet longer lease of life.

"Examinations made from time to time, both by the plate method and direct staining, show conclusively that these spirilla have not only been kept alive, but have also greatly increased in numbers.

"After closely studying the currents of the upper bay, I am led to believe that if dejecta from cholera patients should be thrown into the lower bay, cholera could gain a foothold on the contiguous shores, where every condition favorable to its development and propagation sometimes exists."

This report of Dr. Kinyoun, which appears to have been based upon thoroughly scientific methods of investigation, is not very reassuring to those who have hoped that this country would escape Asiatic cholera, which has already passed through southern Europe, appeared on both shores of South America, and has been arrested in New York harbor by the vigilance of our quarantine officers. If, as many believe, Asiatic cholera is propagated by means of micro-organisms, then it is not only possible, but even probable, that this dread pestilence is now nursing its strength in the waters of New York bay, only to break out upon the surrounding cities as soon as the hot weather appears, enabling it to make its usual inland journeys.

---

A CORRESPONDENT asks the *Medical and Surgical Reporter* for the proper pronunciation of *itis* in gastritis, hepatitis, etc., and the editor replies that either *eyetis* or *etis* is correct, depending upon the system of Latin pronunciation adopted by the speaker. As 999 medical men out of every 1,000 pronounce the technical terms of their profession according to English methods, they should, in order to be consistent, say *eyetis*. If you adopt the Roman or Continental methods of pronunciation, you may call *bronchitis*, *bron-keetis*, but you should, in that case, also pronounce *vaginitis*, *wah-ghe-neetis*, *femur*, *famoor*, and *caecitis*, *kykeetis*, etc. A few years ago, a teacher of anatomy in one of our eastern colleges attempted to apply his so-called Roman method of pronunciation to anatomical words, but only succeeded in making himself extremely ridiculous. We should look upon our technical words, borrowed or manufactured from the classical languages, as English words, and give them an English sound, since we know very little about the ancient pronunciation of Latin and Greek. Every nation in

Europe, except the English, pronounces Latin according to the sounds of the letters in its own language. The French would call Cicero, *seesaro;* the Spanish, *theeharo;* the Italians, *chicharo;* the Germans, *tsitsaro.* Why then should the English, of all the civilized nations using Latin, give words borrowed from this language a foreign sound, or attempt to restore a pronunciation of which they know nothing? We, therefore, advise our readers to pronounce Latin according to English methods, and give the *i* of *itis* its long English sound, thus maintaining a consistent system of orthoepy.

---

THE profession has again been placed under obligations by that reliable and enterprising firm of Philadelphia, Messrs. John Wyeth & Brother. We desire to call attention to the advertisement of their compressed tablet triturates in this issue of our journal. This series of preparations has excited a good deal of attention lately, as the triturates combine so many advantages, are so convenient, so accurate, and those made by this house are prepared with such exactness and beauty of finish, as to commend them to every practitioner. For convenience in prescribing very potent and poisonous drugs, they are invaluable. Their ready solubility and the many admirable combinations embraced in their list, have already made them essential to thousands of our best medical men. We think every physician will read their circular with interest and profit. Their list embraces almost every drug possible to be manufactured in the form of triturates, and the claims made for their therapeutic value are so well founded and confirmed by experience and actual results, no one at all familiar with them can ignore their worth and great importance in our armamentarium. The Messrs. Wyeth will gladly send samples and circular matter to any of our friends who will advise them.

